# 2626. Natural History of RSV Infection in Outpatient Episodes Among Adults in the United States using an EHR Data Source by COVID-19 Era

**DOI:** 10.1093/ofid/ofad500.2239

**Published:** 2023-11-27

**Authors:** Diana Garofalo, Joshua T Swan, Emily Webber, Sally R Omidvar, Margaret Tawadrous, Glenn Pixton, Stephen E Schachterle, Suzanne Landi, Sudeepta Aggarwal, Anindita Banerjee, Niki Alami, Scott P Kelly

**Affiliations:** Pfizer, New York, New York; Pfizer, New York, New York; Truveta, Beaverton, Oregon; Truveta, Beaverton, Oregon; Pfizer, Inc, Groton, Connecticut; Pfizer, New York, New York; Pfizer Inc., Brooklyn, New York; Pfizer, New York, New York; Pfizer, New York, New York; Pfizer, New York, New York; Pfizer, New York, New York; Pfizer, New York, New York

## Abstract

**Background:**

Prevention of severe respiratory syncytial virus (RSV) disease in people at high risk for severe RSV infection (older adults ≥65 years, adults with chronic lung disease, heart failure [HF], immunocompromised) is a significant unmet medical need. Data are sparse on the natural history (including healthcare utilization and progression to severe disease) of the infection in patients initially managed in the outpatient setting. To address this gap, the natural history of RSV infection in outpatient adults was examined in a real-world electronic health record (EHR) data source with daily data updates, enabling analysis of the current season.

**Methods:**

In US-based Truveta EHR database, adults with a positive RSV laboratory test result (real-time PCR or antigen) in the outpatient setting (emergency department (ED), office/clinic, or urgent care) were identified and excluded if immediately hospitalized (within 24 hours of index). To accurately capture preexisting comorbidities, patients were required to have at least one healthcare encounter in the 6 months prior to index. Analyses were stratified by time: pre-COVID-19 (2017-2019), COVID-19 (2020-2021), and current season (2022-January 2023) and restricted to health systems participating for the entire study period. Occurrences of all-cause hospitalization, ED visits, and pneumonia were assessed during 28 days of follow-up (index not included), stratified by risk factors (age ≥65, chronic obstructive pulmonary disease, asthma, or HF).

**Results:**

Among the 6 health systems, 5,807 adults were identified from 2017-2023. Mean ages (standard deviation) were as follows: 52 (20) years (2017-2019); 52 (20) years (2020-2021); 62 (18) years (2022-January 2023). Hospitalization proportions ranged from 7.2-13.2% for all older adults (≥65 years) and were approximately 50% lower when restricting to older adults without other risk factors (Table 1). Expanding to adults 18+ years with risk factors brought hospitalization proportions to 5.7-10.4% (Table 1).

**Figure:**
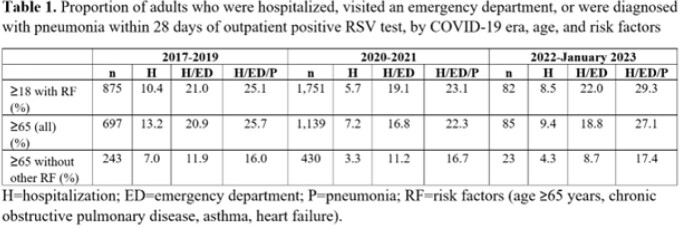

**Conclusion:**

The proportion of high risk adults hospitalized ranged from 3.3-13.2%, with substantial variation by period. Future analyses will include expansion of the study period to include the entire 2022-2023 season and validation in other EHRs in the US.

**Disclosures:**

**Diana Garofalo, PhD MPH**, Pfizer: Stocks/Bonds **Joshua T. Swan, PharmD, MPH, BCPS, FCCM**, CareDx: Grant/Research Support|Genentech: Grant/Research Support|Grifols Share Services North America: Grant/Research Support|Heron Therapeutics: Grant/Research Support|Kedrion Biopharma: Advisor/Consultant|Kedrion Biopharma: Grant/Research Support|Pacira Pharmaceuticals: Grant/Research Support|Pfizer: Grant/Research Support|Pfizer: Employee|Pfizer: Stocks/Bonds|VigiLanz Corporation: Grant/Research Support **Margaret Tawadrous, MD, MS**, Pfizer: Full time employee|Pfizer: Full-time employee|Pfizer: Full-time employee|Pfizer: Full -time employee|Pfizer: Ownership Interest|Pfizer: Ownership Interest|Pfizer: Ownership Interest|Pfizer: Ownership Interest|Pfizer: Stocks/Bonds|Pfizer: Stocks/Bonds|Pfizer: Stocks/Bonds|Pfizer: Stocks/Bonds **Glenn Pixton, MS**, Abbott: Stocks/Bonds|Abbvie: Stocks/Bonds|Pfizer: employee|Pfizer: Stocks/Bonds|Viatris: Stocks/Bonds **Stephen E. Schachterle, Pfizer Inc.**, Pfizer Inc.: Full time employee|Pfizer Inc.: Ownership Interest|Pfizer Inc.: Stocks/Bonds **Suzanne Landi, PhD**, Pfizer, Inc: Employment|Pfizer, Inc: Stocks/Bonds **Sudeepta Aggarwal, Ph.D**, Pfizer: Employee of and may hold stock/stock options in **Anindita Banerjee, Phd**, Pfizer: Employee and stockholder **Niki Alami, MD**, Pfizer: Employee|Pfizer: Stocks/Bonds **Scott P. Kelly, PhD**, Pfizer: Employee|Pfizer: Stocks/Bonds

